# Supplementary figures and images for: Social distancing with chronic pain during COVID-19: A cross-sectional correlational analysis
Source: PLoS One. 2022 Nov 21;17(11):e0275680. doi: 10.1371/journal.pone.0275680 (PMC9678271; doi:10.1371/journal.pone.0275680)

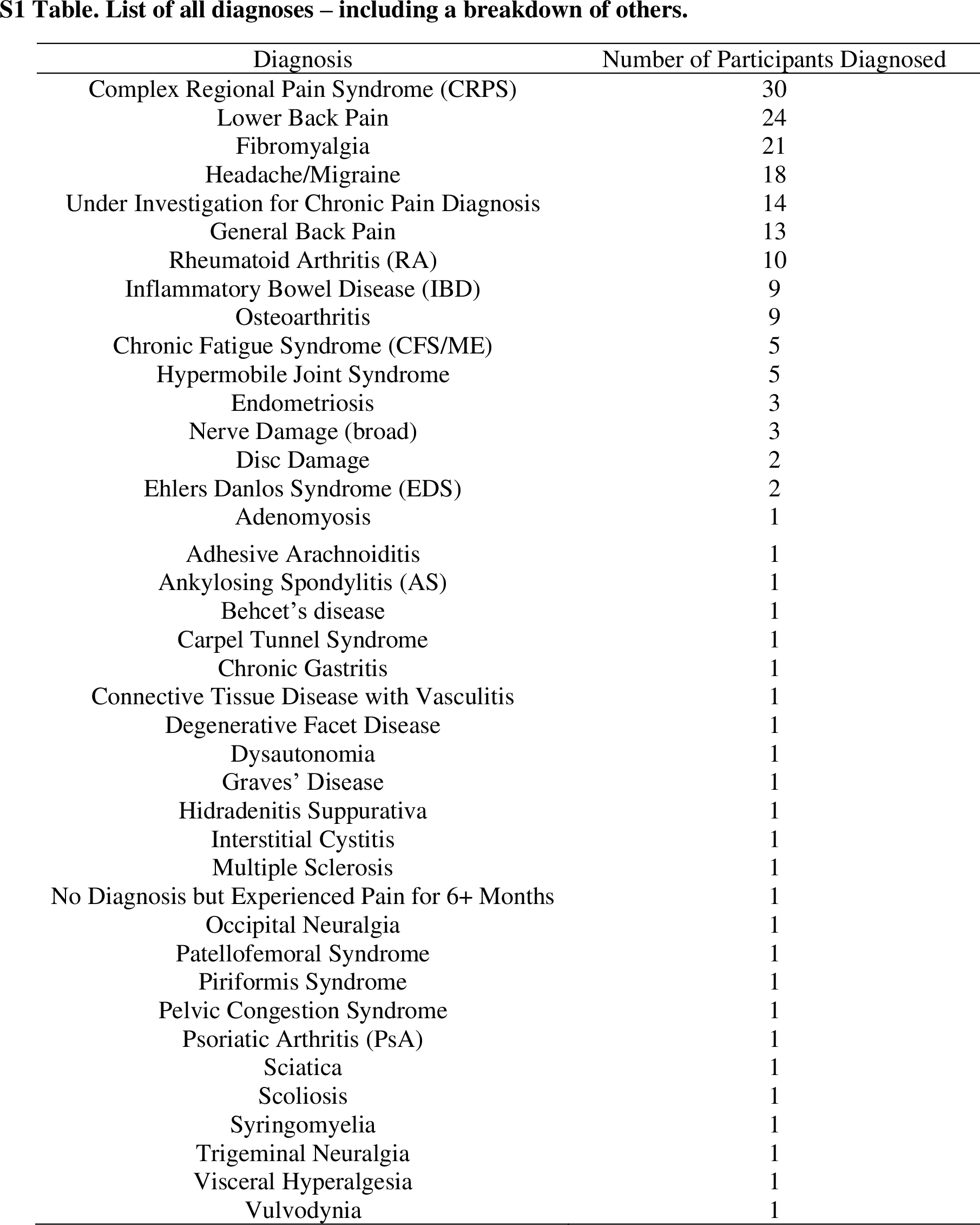

Supplement: S1 Table — (TIF) [file pone.0275680.s001.tif]
